# Risk prediction model of cognitive performance in older people with cardiovascular diseases: a study of the National Health and Nutrition Examination Survey database

**DOI:** 10.3389/fpubh.2024.1447366

**Published:** 2025-01-13

**Authors:** Hui Wang, Sensen Wu, Dikang Pan, Yachan Ning, Cong Wang, Jianming Guo, Yongquan Gu

**Affiliations:** ^1^Department of Vascular Surgery, Xuanwu Hospital, Capital Medical University, Beijing, China; ^2^Department of Intensive Care Medicine, Xuanwu Hospital, Capital Medical University, Beijing, China

**Keywords:** cognitive impairment, older adults, Alzheimer’s disease, nomogram, prediction model, NHANES

## Abstract

**Background and aim:**

Changes in cognitive function are commonly associated with aging in patients with cardiovascular diseases. The objective of this research was to construct and validate a nomogram-based predictive model for the identification of cognitive impairment in older people suffering from cardiovascular diseases.

**Methods and results:**

This retrospective study included 498 participants with cardiovascular diseases aged >60 selected from the NHANES 2011–2014. The study employed the Minor Absolute Shrinkage and Selection Operator (LASSO) regression model, in conjunction with multivariate logistic regression analysis, to identify relevant variables and develop a predictive model. We used statistical techniques as in the Minor Absolute Shrinkage (MAS) and the Selection Operator (LASSO) regression model, in conjunction with multivariate logistic regression analysis, to identify variables that were significantly predictive of the outcome. After which, based on the selected relevant variables, we developed a machine learning model that was predictive of cognitive impairment such as Alzheimer’s diseases in the older people. The effectiveness of the resultant nomogram was evaluated by assessing its discriminative capability, calibration, and conducting decision curve analysis (DCA). The constructed predictive nomogram included age, race, educational attainment, poverty income ratio, and presence of sleep disorder as variables. The model demonstrated robust discriminative capability, achieving an area under the receiver-operating characteristic curve of 0.756, and exhibited precise calibration. Consistent performance was confirmed through 10-fold cross-validation, and DCA deemed the nomogram clinically valuable.

**Conclusion:**

We constructed a NHANES cardiovascular-based nomogram predictive model of cognitive impairment. The model exhibited robust discriminative ability and validity, offering a scientific framework for community healthcare providers to assess and detect the risk of cognitive decline in these patients prematurely.

## 1 Introduction

With the rise in life expectancy, the global population of older people is expanding. Cognitive impairments are becoming increasingly prevalent among the primary concerns in geriatric medicine. Data indicates that from 2000 to 2010, there was a 15% increase in the United States population aged 65 and over, and a 30% increase in those aged 85 and above (1, 2). Age-related cognitive decline represents a substantial health challenge for the older people and cognitive health has emerged as a major public health concern in the aging United States population (3). In the United States, an estimated 36% of the population is affected by cognitive impairment, and there are 5.1 million individuals diagnosed with dementia—a figure projected to double by the year 2050 (4). The economic impact of dementia now surpasses that of cardiovascular and cancer diseases in terms of cost (5, 6). In 2015, the global economic burden of dementia was estimated at $957.56 billion, with projections suggesting an increase to $2.54 trillion by 2030 and further to $9.12 trillion by 2050 (7, 8). The irreversible nature of dementia, coupled with the absence of effective treatments and the substantial economic burden it imposes, underscores the critical importance of preventing and managing cognitive impairment.

In 1982, The Lancet editorial first introduced the term ‘cardiogenic dementia,’ defined as cognitive decline observed in patients following cardiovascular diseases (9). Cardiovascular diseases are commonly linked to atherosclerosis, a condition that affects the medium and large arteries, such as the carotid artery, aorta, and cerebral vessels, leading to reduced blood flow to the brain and subsequent cognitive impairment (10, 11). The prevalence of cardiovascular disease escalates with advancing age, particularly among individuals aged 60 and above. Owing to diminished cognitive reserves, the older people are more susceptible to cognitive impairment following cardiovascular events (12, 13). Furthermore, research has identified that fundamental demographic variables, including depression, educational attainment, diabetes, and income level, are significant factors associated with cognitive impairment (14–17). Cognitive impairment in older patients is an etiologically multifaceted condition, influenced by an interplay of numerous risk factors, rather than a single determinant. Consequently, it is crucial to develop a comprehensive predictive model that integrates all potential risk factors to ascertain the stroke risk within populations. Such a model is pivotal for the early and precise identification of stroke risk, facilitating the timely implementation of suitable preventive measures.

Nomograms serve as predictive instruments that integrate multiple predictors into a graphical representation of statistical models, thereby offering a calculated probability for the occurrence of a clinical event or a specific endpoint outcome (18, 19). Therefore, the development of a nomogram aids in predicting the probability of cognitive impairment, offering timely and tailored preventive recommendations for each individual’s condition. Currently, although other researchers have also conducted studies using machine learning models to predict cognitive impairment but there is a scarcity of clinical predictive models for assessing the risk of cognitive impairment in patients with cardiovascular conditions.

This study employed data from the National Health and Nutrition Examination Survey (NHANES) to develop and validate a nomogram aimed at estimating the risk of cognitive impairment among older patients with cardiovascular disease in the United States. NHANES is a research initiative that assesses the health and nutritional status of American adults and children. The survey utilizes complex, stratified, multistage sampling designs to evaluate the health of the American population. The survey protocol received authorization from the National Center for Health Statistics Research Ethics Review Committee, and all participants completed informed consent forms. All procedures complied with relevant guidelines and regulations. Relevant raw data were meticulously selected from the NHANES database across four key dimensions: demographic information, anthropometric data, laboratory results, and questionnaire feedback. We employed statistical techniques, including the Minor Absolute Shrinkage (MAS) and the Selection Operator (LASSO) regression model, alongside multivariate logistic regression analysis, to identify variables that significantly predicted the outcome. Subsequently, we developed a machine learning model based on these relevant variables to predict cognitive impairment, including conditions like Alzheimer’s disease, in the older people. The model can serve as a reference tool to help Chinese clinicians identify older cardiovascular patients at high risk of cognitive impairment and provide personalized interventions.

## 2 Methods

### 2.1 Study population and data selection

**Figure 1:**
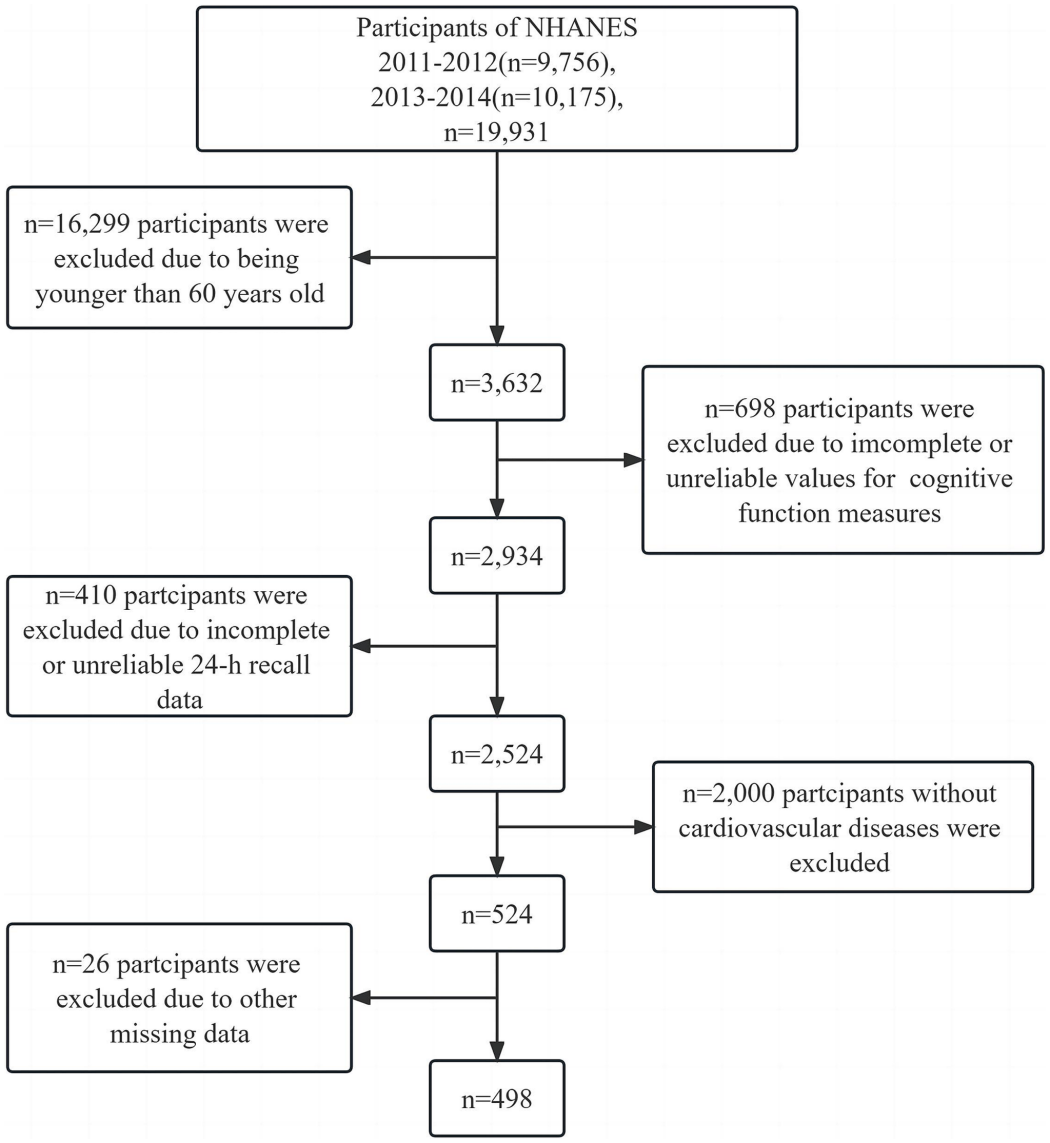
Flow chart of the screening process for the selection of eligible participants.

Our study utilized data from the 2011 to 2014 NHANES^1^ (20). The study excluded data pertaining to pregnant women, cancer patients, and individuals below 60 years of age from the initial participant pool of 19,931. Subjects under 60 years old (*n* = 16,299), participants with unreliable cognitive function test results (*n* = 698), and those with incomplete or inconsistent 24-h dietary recall data (*n* = 410) were omitted from the analysis. Patients without a history of cardiovascular diseases (*n* = 2,000) were excluded from the study. Additionally, participants with substantial missing data (*n* = 26) were excluded; this criterion encompassed individuals with over 80% missing data in any aspect of their demographic information, body measurements, laboratory results, or questionnaire responses. Consequently, the final cohort comprised 498 individuals who had both cardiovascular illnesses and cognitive impairment. A flowchart illustrating the participant selection process is presented in Figure 1. The cardiovascular diseases were defined as self-reported diagnoses made by medical professionals, including (1) heart failure, (2) coronary heart disease, (3) angina, (4) myocardial infarction, and (5) stroke.

### 2.2 Ethical approval

The authors bear responsibility for all aspects of this research, including addressing inquiries regarding the accuracy and integrity of any component, and resolving them appropriately. The study was conducted in accordance with the revised 2013 Declaration of Helsinki. The study involving participants were reviewed and approved through the NHANES has been approved through the National Center for Health Statistics Research Ethics Review Board. Prior to participating in the study, all participants provided written informed consent.

### 2.3 Assessment of cognitive performance

During the investigation conducted between 2011 and 2014 as part of the NHANES study, participants underwent a battery of cognitive assessments (21). The Consortium to Establish a Registry for Alzheimer’s Disease (CERAD) Word List Learning subtest, Animal Fluency test (AFT), and Digit Symbol Substitution Test (DSST) were employed for the evaluation of cognitive abilities within a Mobile Examination Center (MEC). These assessments are specifically designed to gauge various cognitive domains, including working memory, language proficiency, processing speed, and overall cognitive function among older adults. They have found widespread application in screening, epidemiological investigations, and clinical research (22–26). With the participants’ informed consent, the testing procedures were audio-recorded to ensure the quality of data collection. This necessitated the use of a dual interviewing technique, with assessments for the CERAD, AT, and DSST conducted in both Spanish and English. Subsequently, responses were transcribed and evaluations were assigned based on the written content, typically completed on the same day. Assessments conducted in languages other than English or Spanish were meticulously scored by experts who transcribed the responses. In cases where score discrepancies arose, an impartial adjudicator was consulted to make a determination. During the data collection process, approximately 10% of the interview recordings underwent independent review (27).

The CERAD test assesses both immediate and delayed learning of new linguistic material. It comprises three consecutive learning trials followed by a delayed recall trial. During the learning trials, each participant was presented with ten unrelated words to articulate one at a time. Participants were then prompted to recollect as many words as possible immediately after the word presentation. Delayed word recall occurred subsequent to DSST testing and animal fluency testing. The CERAD test score is the sum of the three learning trials and one delayed recall trial, with each trial rated on a scale from 0 to 10 (27, 28). The AFT evaluates category-based language fluency as an aspect of executive functioning by instructing participants to list as many animals as possible within 1 min. The total count of correct responses constitutes the score (29). The DSST performance component of the Wechsler Adult Intelligence Scale assesses working memory, sustained attention, and processing speed. Participants are tasked with completing this assessment using a paper sheet containing 133 boxes, each pairing a number with a corresponding symbol. Within a two-minute time frame, participants are required to accurately replicate the corresponding symbol for each number. The final score, ranging from 0 to 133, is based on the number of correct matches (30, 31).

As of the present, no definitive benchmarks exist for identifying below-average cognitive performance in the DSST, CERAD, or AFT. Therefore, following the approach commonly employed in existing literature, we established the threshold at the 25th percentile, which corresponds to the lowest quartile of scores (32). Furthermore, considering the significant impact of age on cognitive function, the scores were stratified into distinct age groups: 60 to <70 years, 70 to <80 years, and ≥ 80 years (8, 33). The threshold scores denoting low cognitive abilities on the CERAD test were 17, 20, and 21 for the respective age groups. In the case of the AFT, the thresholds were 13, 12, and 11; whereas for the DSST, they were 33, 26, and 27. Participants were divided into two categories for each dimension: individuals scoring below the respective threshold were categorized into the low cognitive performance group, while the remaining participants were assigned to the normal cognitive performance group.

### 2.4 Assessment of covariates

Demographic information regarding the participants was collected through a self-administered questionnaire. The demographic characteristics were classified based on gender (male, female), race (Mexican American, non-Hispanic white, non-Hispanic black, Hispanic, other race), marital status (unmarried, married, cohabiting, separated, divorced, or widowed), and educational attainment. Educational levels in our study were categorized as follows: less than high school (below 9th grade), high school education (including 9th-12th grade, GED, or equivalent), or college or higher (some college, Associate’s degree, or college graduate and above). Individuals who reported having smoked fewer than 100 cigarettes in their lifetime were categorized as never smokers. Current smokers were defined as those who had smoked more than 100 cigarettes during their lifetime, while former smokers were individuals who had smoked more than 100 cigarettes but had subsequently quit smoking. Poverty income ratio (PIR) scores were categorized as less than 1, 1–3, and more than 3. PIR is calculated by dividing household income by the poverty guidelines specific to the survey year (34, 35).

### 2.5 Statistical analysis

All statistical analyses were conducted using R software (version 4.3.1). The dataset from the NHANES database was randomly divided into training and validation sets in a 7:3 ratio for variable comparison. Non-normally distributed data are presented as median values with interquartile ranges. Categorical data are reported as frequencies and percentages, and univariate analysis utilized Fisher’s exact test or the chi-square test. For continuous variables reported as means and standard errors, the rank sum test or t-test was applied. In the context of linear regression modeling with shrinkage and variable selection, we have applied the Least Absolute Shrinkage and Selection Operator (LASSO) regression technique. Initially, we employed the LASSO regression method to assess the dataset using the development set data. Before performing LASSO regression, all input features were standardized to ensure they had similar scales. K-fold cross-validation was used to select the regularization parameter *λ* for LASSO regression, and the selected λ value was then used to train the LASSO regression model. Subsequently, based on lambda, we selected five independent variables for LASSO regression analysis. This analysis aimed to identify significant and effective risk predictors for patients with both cognitive impairment and cardiovascular disorders. After identifying the key predictors, a multivariable logistic regression model was constructed using these selected variables. A nomogram was then created based on this model to visually represent the contribution of each predictor to the risk of cognitive impairment. The nomogram provides an individualized risk estimate by assigning scores based on the impact of each predictor, and the total score derived from these scores gives an overall risk assessment. Subsequently, we employed the results obtained from logistic regression analysis to construct three distinct models: the stepwise (stepAIC) selected model, the full model, and the multiple fractional polynomial (MFP) model. To characterize the features, we utilized odds ratios and corresponding *p*-values, providing a 95% confidence interval (CI). Simultaneously, the selection of the model with the highest area under the curve (AUC) significance was based on a comparison of each model’s receiver operating characteristic (ROC) curve in both the development and validation datasets. Additionally, we assessed model consistency using the Hosmer-Leme show test and calibration curve. Decision curve analysis (DCA) was employed to evaluate the model’s clinical efficacy. All statistical analyses were two-sided, with a significance level set at alpha = 0.05.

## 3 Results

### 3.1 Characteristics of participants

The demographic characteristics of the study population are detailed in Table 1. A total of 498 participants met the predefined inclusion and exclusion criteria for this study. Regarding hypertension, a greater proportion of participants in the training cohort had hypertension (267 out of 346, 77%) compared to the validation cohort (127 out of 149, 85%), and this disparity was statistically significant (*p* = 0.028). Conversely, other characteristics such as age group, race, smoking status, diabetes status, high cholesterol, depressive symptoms, sleep disorders, and aspirin usage did not exhibit significant differences between the two cohorts.

**Table 1:** Characteristics of study participants.

Characteristic	Training cohort, *N* = 349^1^	Validation cohort, *N* = 149^1^	*p*-value^2^
Gender			0.518
Female	146 (42%)	67 (45%)	
Male	203 (58%)	82 (55%)	
Age			0.284
60–69	138 (40%)	55 (37%)	
70–79	111 (32%)	58 (39%)	
≥80	100 (29%)	36 (24%)	
Race			0.332
Mexican American	25 (7.2%)	8 (5.4%)	
Other Hispanic	61 (17%)	33 (22%)	
Non-Hispanic white	23 (6.6%)	14 (9.4%)	
Non-Hispanic black	215 (62%)	80 (54%)	
Other race	25 (7.2%)	14 (9.4%)	
Education level			0.069
Less than 9th grade	36 (10%)	25 (17%)	
9-12th grade	51 (15%)	26 (17%)	
College or above	262 (75%)	98 (66%)	
Marital status			0.716
Married	192 (55%)	78 (52%)	
Widowed	75 (21%)	39 (26%)	
Divorced	53 (15%)	17 (11%)	
Separated	8 (2.3%)	4 (2.7%)	
Unmarried	16 (4.6%)	8 (5.4%)	
Living with a partner	5 (1.4%)	3 (2.0%)	
Poverty income ratio			0.280
1	70 (20%)	36 (24%)	
2	169 (48%)	76 (51%)	
3	110 (32%)	37 (25%)	
Smoke			0.672
Never smokers	145 (42%)	68 (46%)	
Current smokers	152 (44%)	59 (40%)	
Former smokers	52 (15%)	22 (15%)	
Diabetes			0.883
Yes	143 (41%)	60 (40%)	
No	206 (59%)	89 (60%)	
Hypertension			0.028
Yes	267 (77%)	127 (85%)	
No	82 (23%)	22 (15%)	
High Cholesterol			0.274
Yes	248 (71%)	113 (76%)	
No	101 (29%)	36 (24%)	
Depression			0.060
Yes	280 (80%)	130 (87%)	
No	69 (20%)	19 (13%)	
Sleep disorder			0.394
Yes	75 (21%)	27 (18%)	
No	274 (79%)	122 (82%)	
Use of aspirin			0.139
Yes	257 (74%)	119 (80%)	
No	92 (26%)	30 (20%)	
ALB	41.3 ± 3.0	41.5 ± 3.2	0.489
ALT	23 ± 15	21 ± 12	0.198
AST	26 ± 14	26 ± 13	0.993
Alkaline Phosphatase	69 ± 26	74 ± 24	0.060
Urea Nitrogen	19 ± 10	19 ± 8	0.392
Serum Calcium	2.36 ± 0.10	2.34 ± 0.09	0.017
Creatine Kinase	121 ± 93	125 ± 99	0.657
Serum Creatinine	104 ± 52	113 ± 73	0.177
Total Cholesterol	171 ± 42	171 ± 47	0.904
Fasting Blood Glucose	6.54 ± 2.29	7.00 ± 3.71	0.163
Serum iron	14.3 ± 6.1	14.2 ± 6.1	0.856
Serum phosphorus	1.23 ± 0.19	1.22 ± 0.19	0.430
TBIL	11.4 ± 4.9	12.8 ± 4.6	0.004
Uric Acid	6.12 ± 1.65	6.15 ± 1.57	0.832
Triglycerides	170 ± 118	153 ± 88	0.081
Glycated hemoglobin	6.31 ± 1.08	6.46 ± 1.59	0.289
HDL	51 ± 17	49 ± 13	0.235

1 *n* (%).

2 Pearson’s Chi-squared test; Fisher’s exact test; Welch Two Sample t-test.

ALT, alanine aminotransferase; AST, aspartate aminotransferase; HDL, High-density lipoprotein; TBIL, total bilirubin; ALB, albumin.

### 3.2 Predictive model

**Figure 2:**
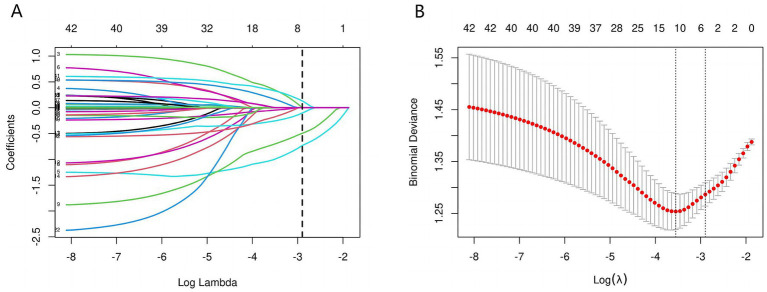
Predictors’ selection using the Lasso regression method. As shown in the figures, during the LASSO regularization process, the regression coefficients of various features exhibit a trend of gradually approaching zero or stabilizing along the log(*λ*) axis **(A)**. When λ is small (left side), the model retains a larger number of nonzero coefficients, yet some feature weights display substantial variability. As λ increases, the coefficients gradually shrink toward zero, leaving only relatively stable and influential variables with nonzero coefficients. Concurrently, the cross-validation-based error analysis **(B)** reveals that the Binomial Deviance reaches its minimum at around log(λ) = −4, indicating the model’s optimal predictive performance. The dashed lines mark the empirically determined optimal range of λ, providing an objective basis for balancing model complexity with predictive accuracy. Within this range, the model’s coefficients are notably simplified and the deviance is significantly reduced, thereby achieving improved predictive performance and enhanced robustness.

The initial model included a multitude of candidate predictors in the fields of sex, age group, race, education level, marital status, ALB, PIR, ALT, AST, alkaline phosphatase, urea nitrogen, serum calcium, CPK, TC, fasting plasma glucose, creatinine, serum iron, serum phosphorus, total bilirubin, uric acid, TG, smoking status, diabetes, stroke, hypertension, high cholesterol, glycosylated hemoglobin, HDL, depression, sleep disorder, and aspirin use. However, employing LASSO regression analysis within the training cohort, the model was subsequently refined, resulting in the identification of five potential predictors. Figure 2A displays the coefficient profile, while Figure 2B presents a cross-validated error plot of the LASSO regression model. The most parsimonious model, which maintained a cross-validated error within one standard error of the minimum, incorporated five variables (age, race, educational level, sleep disorder and PIR). We performed multivariable logistic regression on these five factors, and the results showed that they had statistically significant differences (Table 2).

**Table 2:** Multivariate logistic regression of covariates associated with cognitive impairment in older cardiovascular patients.

Variables	OR	95%CI	*p*-value
Age			
≥80	(Reference)		
70–79	0.89	(0.53 ~ 1.03)	0.623
60–69	0.73	(0.45 ~ 0.96)	0.049
Race			
Non-Hispanic black	(Reference)		
Other race	0.39	(0.17 ~ 0.91)	0.029
Non-Hispanic white	0.25	(0.11 ~ 0.59)	0.001
Mexican American	0.16	(0.05 ~ 0.44)	<0.001
Other Hispanic	0.29	(0.16 ~ 0.51)	<0.001
Education level			
Less than 9th grade	(Reference)		
9-12th grade	0.87	(0.64 ~ 1.05)	0.039
College or above	0.60	(0.38 ~ 0.94)	<0.001
PIR			
1	(Reference)		
2	1.45	(0.82 ~ 2.57)	0.201
3	2.76	(1.47 ~ 5.18)	0.002
Sleep disorder			
Yes	(Reference)		
No	0.60	(0.35 ~ 0.92)	0.041

OR, Odds Ratio; CI, Confidence Interval.

### 3.3 Development and validation of the nomogram

**Figure 3:**
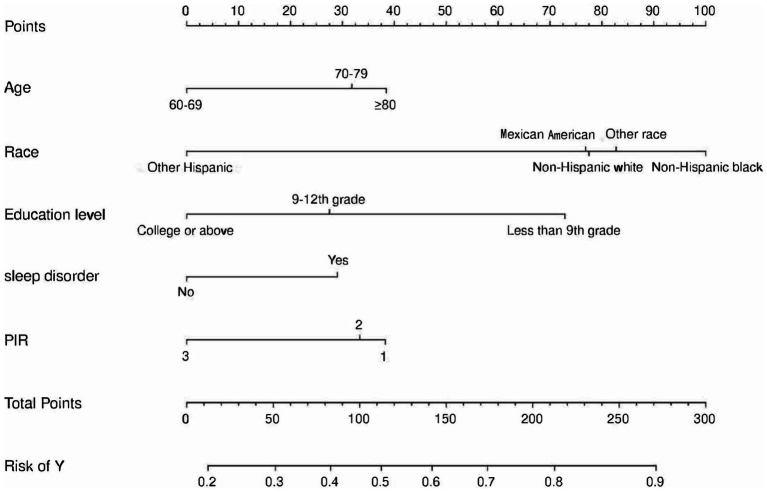
Nomogram for prediction of cognitive impairment risk and its predictive performance. The cognitive impairment risk nomogram was developed with the predictors including age, race, PIR, education level, and sleep disorder. PIR, Poverty income ratio.

**Figure 4:**
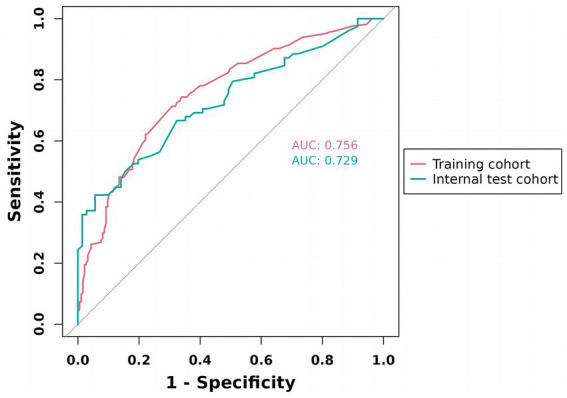
ROC validation of the cognitive impairment risk nomogram prediction. ROC, receiver-operating characteristic. As shown, the model achieved AUCs of 0.756 (training) and 0.729 (internal test), both exceeding 0.7, indicating good discriminative ability and stable generalization.

**Figure 5:**
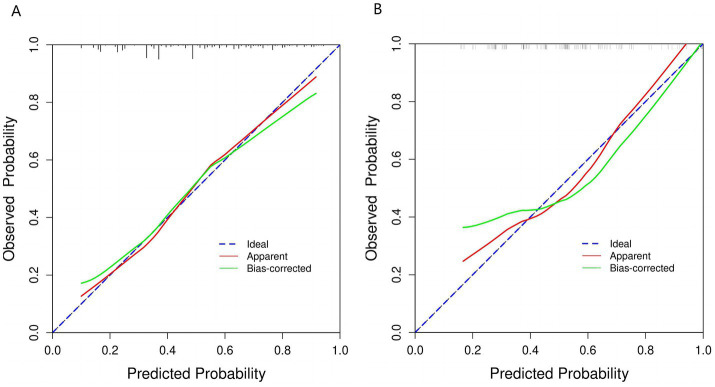
Calibration curve of the nomogram prediction mode. **(A)** Calibration curve for training cohort. **(B)** Calibration curve for validation cohort. This figure shows that the model’s calibration curve deviates from the ideal line (blue dashed line). The original result (red line) departs from the ideal, but after bias correction (green line), it aligns more closely with the ideal state. This indicates improved consistency between predicted probabilities and actual incidence, suggesting enhanced calibration performance.

We constructed a comprehensive predictive nomogram comprising age, educational attainment, racial background, presence of sleep disorders, and poverty income ratio. This nomogram was developed by applying the minimum criteria, including non-zero coefficients from Lasso regression and significant findings from logistic regression screening. Each predictor was quantified as a specific score on a rating scale. The cumulative scores for all variables were summed, and a vertical line was drawn downwards on the scale, corresponding to the probability of cognitive impairment. A higher score on the scale indicates an elevated likelihood of cognitive impairment (as illustrated in Figure 3). ROC curves were employed to assess the discriminative performance of the model on both the training and test sets. The ROC analysis yielded an AUC of 0.756 for the training set and an AUC of 0.729 for the validation set, demonstrating the model’s robust stability and predictive accuracy. These results are graphically presented in Figure 4. Additionally, calibration curves, generated through 1,000 bootstraps, exhibited a strong alignment between actual and predicted probabilities within the nomogram, affirming the stability and precision of the predictive models (Figure 5).

### 3.4 Decision curve analysis

**Figure 6:**
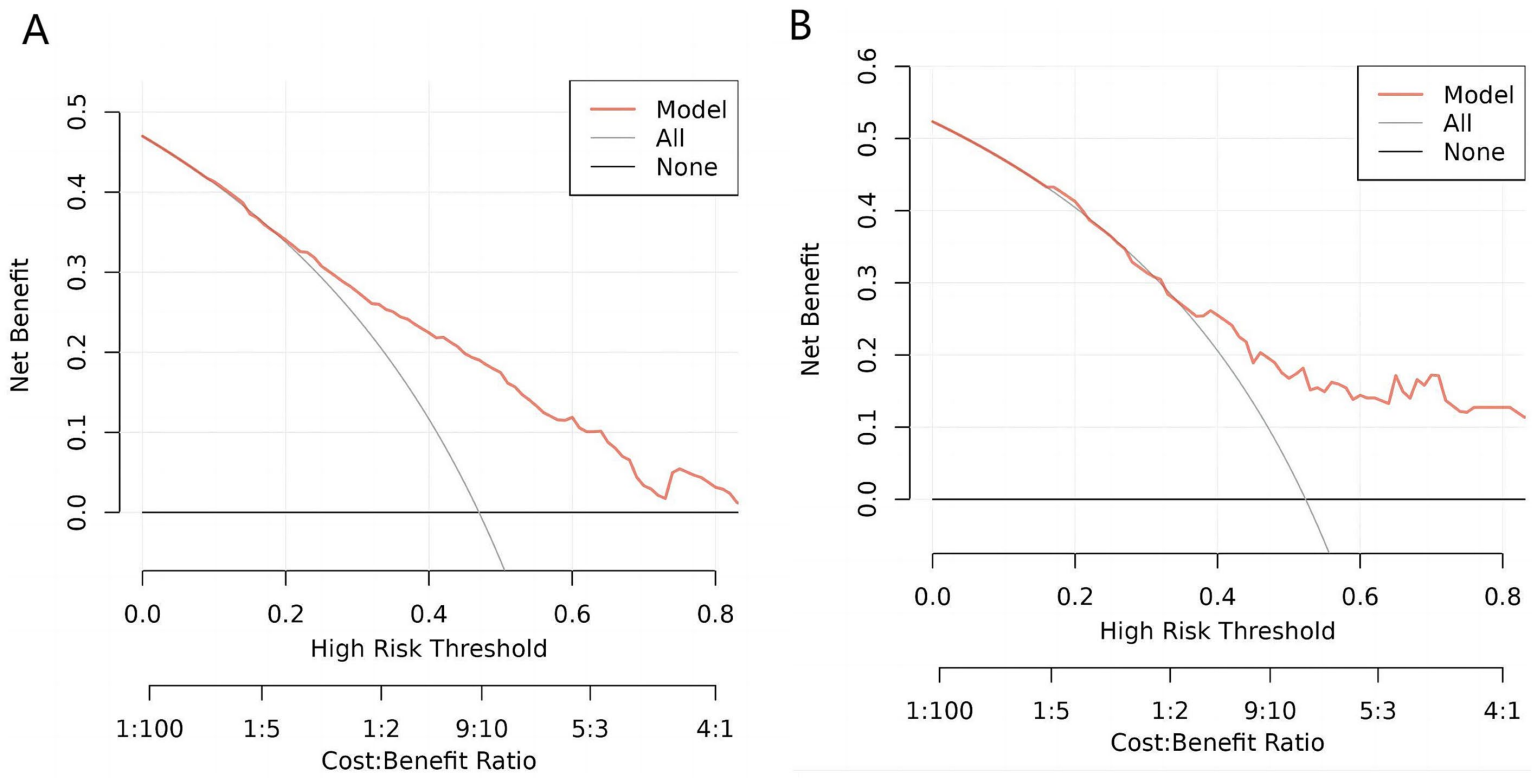
Decision curve analysis (DCA) of the nomogram. **(A)** DCA for training cohort. **(B)**. DCA for validation cohort. This decision curve demonstrates that, compared to treating all patients (gray line) or none (black line), the model (red line) yields a higher net benefit within a certain range of high-risk thresholds. Under appropriate threshold conditions, employing this model can achieve greater clinical net benefit, thereby improving the decision-making process.

Figure 6 presented below, illustrates the DCA curves associated with the nomogram. A high-risk threshold probability signifies the potential for significant disparities in the model’s predictions when clinicians encounter substantial limitations while utilizing the nomogram for diagnostic and decision-making purposes. This study demonstrates that the nomogram provides substantial net benefits for clinical application, as evident from its DCA curve.

## 4 Discussion

Identifying the optimal predictive model for the prevention of cognitive impairment holds clinical significance, given the widespread prevalence of cognitive impairment and the associated high treatment and care costs. Therefore, for older adults diagnosed with cardiovascular diseases, we developed and validated a predictive nomogram for cognitive impairment. The nomogram incorporates five factors: age, race, educational attainment, poverty income ratio, and sleep disturbances. All of these independent predictors within the nomogram are readily accessible and demonstrated favorable results in internal validation.

Our study encompasses the inclusion of all older people with cardiovascular issues. In comparison to their counterparts among senior citizens, this group exhibits a heightened susceptibility to cognitive impairment attributed to their elevated risk factors for cerebral and intracranial vascular atherosclerosis. The current study’s findings underscore age as a significant prognostic indicator for the likelihood of cognitive impairment. Previous research has consistently identified age as a prominent risk factor for dementia and cognitive decline (36, 37). Examining the nomogram, it becomes evident that the association between age and the risk of cognitive impairment exhibits a non-linear pattern, in line with findings from prior research (38–40).

Furthermore, our study reveals a robust correlation between educational attainment, poverty index, and the likelihood of cognitive impairment among older people with cardiovascular disease. Individuals with lower income and educational levels exhibit a higher susceptibility to cognitive impairment, and this association is strengthened by both variables. To a certain extent, education contributes to the development of cognitive skills, including logical reasoning and abstract thinking, while also playing a role in the preservation of neurons and the enhancement of cognitive abilities (41). Based on social psychology theory, individuals with higher levels of education tend to have higher incomes and are presumed to possess enhanced coping abilities and problem-solving skills. These attributes may contribute, to some extent, to the deceleration of cognitive decline (42–45).

Moreover, our estimation suggests that non-Hispanic Black patients are likely to exhibit the highest incidence of cognitive impairment compared to other ethnic groups, and they significantly contributed to the prediction of this outcome. This discovery aligns with our research and is supported by demographic data from 2020 for patients in the United States with mild cognitive impairment and Alzheimer’s disease, which indicated that the largest proportion consisted of non-Hispanic Black individuals (46, 47). According to Bubu OM’s meta-analysis (48), individuals experiencing sleep difficulties are 1.68 times more likely to encounter cognitive impairment as a composite outcome (95% CI: 1.51–1.87). These findings hold significance in the context of potential prevention of cognitive decline, given the growing public apprehension regarding sleep-related concerns (49, 50).

In clinical practice, the nomogram model can be used in various scenarios. First, during a patient’s initial visit, the physician can use the nomogram to quickly assess the risk of cognitive impairment. By inputting the patient’s age, race, education level, sleep disorder status, and PIR into the nomogram model, the physician can obtain a quantitative risk score, which helps identify high-risk patients. This risk prediction can assist doctors in taking early interventions, such as recommending further neuropsychological assessments or arranging follow-up monitoring of cognitive function. Additionally, for patients identified as high risk, physicians can reduce their future risk of cognitive impairment by addressing sleep problems, suggesting cognitive training, and providing socioeconomic support. The nomogram can also be used for follow-up management. For patients already identified as high risk, the nomogram can be used at each follow-up to reassess risk levels and track the effectiveness of interventions. This helps to dynamically adjust personalized treatment plans, allowing patients to receive optimal management in the early stages of the disease. Moreover, the nomogram model is simple and easy to use, and it can also be used as an educational tool to explain to patients and their families the impact of different factors on cognitive health, enhancing patients’ awareness of their own health and encouraging active participation. One of the primary strengths of this study lies in the development of a nomogram model characterized by its high degree of generality and user-friendliness. To visually represent the relative significance of predictive factors using segment lengths, we employed a nomogram plot. This approach enabled the conversion of complex regression equations into comprehensible graphical representations. Our diagnostic tool demonstrated outstanding performance in both the training and validation cohorts, exhibiting strong accuracy in both calibration and discriminative capabilities. Additionally, we utilized DCA, a method that quantifies net benefits without necessitating information on treatment costs, effectiveness, or patient preferences regarding different health states. DCA offers insights into clinical implications based on threshold probabilities (51).

Our predictive approach still exhibits several limitations. Firstly, our nomogram model was constructed based on retrospective data from the NHANES database. Consequently, the model’s accuracy was compromised due to the reliance on self-reported variables, which introduced selection bias. Secondly, the cohort may not be representative of the broader population as it was derived from American patients. Furthermore, our model might not have accounted for potential unmeasured confounding variables.

To establish the generalizability of our findings, external validation across diverse populations is essential. First, although our nomogram prediction model was constructed based on data from a specific population, it can theoretically be applied to other similar populations, such as the older people in China. Since certain characteristics in the older people (e.g., changes in age, differences in education levels, insomnia) may be similar across different regions, external validation can help determine whether these similar factors can also influence the model’s predictive performance. Furthermore, in order to generalize this prediction model to populations in other countries, especially in regions with significant differences in population structure (such as Europe, Africa, and South America), it is necessary to consider significant differences in genetic background, environmental factors, lifestyle, and healthcare conditions. These differences may lead to decreased predictive accuracy of the model. Therefore, when developing a nomogram prediction model for these countries, sufficient data should be collected locally for recalibration and validation to ensure the model’s accuracy and reliability. This process can be achieved through retraining the model or adjusting model parameters to adapt to different population characteristics and improve predictive performance.

## 5 Conclusion

In summary, utilizing data sourced from the NHANES database, we developed a nomogram for the prediction of cognitive impairment in older people with cardiovascular diseases. The nomogram incorporates variables such as age, race, educational attainment, sleep disorders, and the poverty income ratio. Our predictive tool exclusively utilizes objective and biologically plausible predictors that are readily available. This innovation holds the potential to improve the quality of life among older individuals with cardiovascular diseases, mitigate the onset and progression of cognitive impairment, and raise physicians’ awareness of early indicators of cognitive decline.

## Data Availability

Publicly available datasets were analyzed in this study. This data can be found here: https://www.cdc.gov/nchs/index.html.

## Footnotes

^1^ https://www.cdc.gov/nchs/index.html
